# Percutaneous coronary revascularization in patients with formerly "refractory angina pectoris in end-stage coronary artery disease" – Not "end-stage" after all

**DOI:** 10.1186/1471-2261-9-42

**Published:** 2009-08-28

**Authors:** Thomas W Jax, Ansgar J Peters, Ahmed A Khattab, Matthias P Heintzen, Frank-Chris Schoebel

**Affiliations:** 1 Profil Institut für Stoffwechselforschung, Hellersbergstrasse 9, 41460 Neuss, Germany; 2 Klinik für Kardiologie, Herzentrum Wuppertal and Institut für Herz-und Kreislaufforschung, Dortmund both Universität Witten-Herdecke, Germany; 3 Klinik für Kardiologie, Universitätsklinikum Düsseldorf, Frank-Chris Schoebel, MD Dr. Ansgar Peters, MD Moorenstrasse 5, 40 225 Düsseldorf; 4 Segeberger Kliniken GmbH, Herz-Kreislauf-Zentrum, Ahmed Khattab, MD Am Kurpark 1, 23 795 Bad Segeberg, Germany; 5 Klinikum Braunschweig, Klinik für Kardiologie, Prof. Matthias Heintzen, MD Salzdalumerstrasse 90, 38 126 Braunschweig, Germany

## Abstract

**Background:**

Patients with refractory angina pectoris in end-stage coronary artery disease represent a severe condition with a higher reduction of life-expectancy and quality of life as compared to patients with stable coronary artery disease. It was the purpose of this study to invasively re-evaluate highly symptomatic patients with formerly diagnosed refractory angina pectoris in end-stage coronary artery disease for feasible options of myocardial revascularization.

**Methods:**

Thirty-four Patients formerly characterized as having end stage coronary artery disease with refractory angina pectoris were retrospectively followed for coronary interventions.

**Results:**

Of those 34 patients 21 (61.8%) were eventually revascularized with percutaneous interventional revascularization (PCI). Due to complex coronary morphology (angulation, chronic total occlusion) PCI demanded an above-average amount of time (66 ± 42 minutes, range 25–206 minutes) and materials (contrast media 247 ± 209 ml, range 50–750 ml; PCI guiding wires 2.0 ± 1.4, range 1–6 wires). Of PCI patients 7 (33.3%) showed a new lesion as a sign of progression of atherosclerosis. Clinical success rate with a reduction to angina class II or lower was 71.4% at 30 days. Surgery was performed in a total of8 (23.5%) patients with a clinical success rate of 62.5%. Based on an intention-to-treat 2 patients of originally 8 (25%) demonstrated clinical success. Mortality during follow-up (1–18 months) was 4.8% in patients who underwent PCI, 25% in patients treated surgically and 25% in those only treated medically.

**Conclusion:**

The majority of patients with end-stage coronary artery disease can be treated effectively with conventional invasive treatment modalities. Therefore even though it is challenging and demanding PCI should be considered as a first choice before experimental interventions are considered.

## Background

Refractory angina pectoris in end-stage coronary artery disease is characterized by severe coronary insufficiency but only moderately impaired left ventricular function [1]. This in comparison to other patients with stable angina pectoris not only leads to a reduced quality of life as a result of invalidating clinical symptoms but also to a severe reduction of life expectancy with a mortality rate of 10 to 15% in the first year following diagnosis [2-5]. According to the definition maximal intensification of conventional medical and invasive treatment modalities have been ruled out as feasible options, hence these patients represent the frontier of clinical science and skill in the treatment of patients with coronary artery disease.

For patients with refractory angina pectoris in end-stage coronary artery disease various alternative therapeutic approaches are of interest and have in part been tested: transmyocardial laser revascularization, spinal cord stimulation, pharmacological und surgical sympathectomy, gene-therapy, bone-marrow transplantation, external counterpulsation and medical treatments long-term intermittent urokinase therapy and trimetazidine [2-12]. As these concepts of indirect revascularization, neurophysiological modulation, microcirculatory and metabolic optimization are more or less successful they do not tackle the root of the problem, the impairment of conductive blood flow at the level of the epicardial coronary arteries. Established interventions like percutaneous coronary interventions (PCI) and coronary artery bypass operation (CABG) which restore conductive blood flow are therefore the most effective for relieve of ischemia and symptoms. In particular PCI but also CABG has seen improvements of materials and skills during recent years which have led to better immediate and long-term results and reduced the technical limitations of revascularization.

Therefore patients who had been previously been diagnosed to suffer from refractory angina pectoris in end-stage coronary artery disease were reevaluated angiographically. It was the therapeutic aim of the study presented to perform primarily PCI or CABG in this high-risk group where feasible.

## Methods

### Patients

For the study 34 consecutive patients were included who had been previously successfully treated with long-term intermittent urokinase therapy, a rheological approach developed for the improvement of the coronary microcirculation [11]. All patients had fulfilled the criteria of refractory angina pectoris in end-stage coronary artery disease prior to the beginning of long-term intermittent urokinase therapy. Refractory angina pectoris in coronary artery disease is defined as the persistence of stable severe anginal symptoms grade III or IV according to the criteria of the Canadian Cardiovascular Society [13]. Further, the option to use an invasive revascularization procedure such as percutaneous coronary balloon angioplasty or aorto-coronary bypass grafting must be excluded by cardiologists and cardiac surgeons on the basis of a recent coronary angiogram [1]. After treatment with 500.000 i.u. urokinase intravenously, three times a week for 12 weeks all patients had improved clinically at least to CCS-grade II and were further monitored in the outpatient department for follow-up. Patients returned for renewed coronary angiography as clinical symptoms had deteriorated again to angina grade III or IV. Coronary angiography was the first invasive procedure in our center scheduled after treatment with long-term intermittent urokinase therapy.

This retrospective observational study was in compliance to the principles of the Declaration of Helsinki. All patients consented in writing to any and especially the interventional procedures as well as to the use of data for scientific use as part of their hospital admission. All diagnostic tests, treatments and interventions were, for the individual patient, "standard care". None of the patients involved in this study received any study specific diagnostic tests or treatments. The use of retrospective data was approved by the ethics committee of Heinrich-Heine-University Düsseldorf, Germany.

### Coronary angiography and percutaneous intervention

Coronary angiograms were evaluated for potential culprit lesions which could be regarded as relevant for myocardial ischemia and hence for the clinical symptoms of the patient. In all patients stress exercise testing for electrocardiographic signs of myocardial ischemia were performed, in some patients additional myocardial stress-scintigraphy was done. These stress tests were mostly inconclusive as almost all patients suffered form severe triple vessel disease and had limited exercise capacity due to clinical symptoms and beta-blocker therapy. Therefore the decision to treat with PCI was mostly based on the experience of two interventional cardiologists (FCS, MPH). Where feasible the decision to treat surgically was made together with cardiac surgeons.

Critical coronary lesions were classified as chronic total occlusions, angulated lesions, and last patent vessels. As part of the invasive work-up of these high-risk patients PCI was usually scheduled for a second session in order to prepare the intervention meticulously. Interventions were performed by two interventional cardiologists (FCS, MPH) either alone or sometimes even alternating in one patient in case one encountered difficulties, for example crossing a chronic total occlusion. In general 7 French guiding catheters (Zuma™, Medtronic, St. Paul, MN, USA) were used for optimal visualization and back-up; various PCI guiding wires, low-profile balloons and stents were used (see also below). Angiography was performed in a biplane technique.

Interventions were regarded as successful when the lesion could be dilated completely or if bypass vessels could be attached surgically. Clinical success was defined as a reduction of clinical symptoms at least to stage II angina pectoris for more than 30 days.

## Results

### Patients

Patients had a mean age of 67 ± 10 years (range 45–85 years), 11.8% were female (Table 1). Time since long-term intermittent urokinase therapy was 26 ± 14 months (range 5–61 months). Of the total group 91.2% presented with coronary triple vessel disease; 82.4% had undergone a previous coronary artery bypass operation, 25% of whom more than one operation.

**Table 1:** Patient Characteristics

	Patients n = 34
Age (yrs)	67 ± 10
Male (%)	82.2
Female (%)	11.8
Height (m)	174 ± 8
Weight (kg)	86 ± 19
	
*Vascular risk factors*	
Arterial hypertension (%)	88.2
Diabetes mellitus (%)	38.2
Insulin dependent of diabetes mellitus total (%)	46.2
Previous smoking (%)	52.9
	
*Medication*	
Nitrate (%)	91.2
Beta blocker (%)	88.2
Calcium antagonist (%)	55.9
Double combination therapy (%)	41.2
Triple combination therapy (%)	44.2%
	
*Prior invasive cardiac treatment*	
PCI (%)	58.8
> 2 PCI of PCI total (%)	45.0
	
CABG (%)	82.4
> 1 CABG of CABG total (%)	25.0

### Treatment

Based on an intention-to-treat the majority of 20 (58.8%) of the 34 patients were treated with PCI, while 6 (17.7%) were referred to surgery. In 8 (23.5%) patients we primarily did not see a reasonable and safe option for mechanical revascularization and these patients were treated medically. For persistence or progression of clinical symptoms 4 patients primarily treated medically, were treated invasively later on, 2 with PCI and 1 with surgery, while 1 patient in the PCI group was switched over to the surgical group.

### Percutaneous coronary intervention

Of the total of 21 patients (61.8% of all patients) eventually treated with PCI an angulated lesion was targeted in 8 (38%), a chronic total occlusion in 6 (29%) and the last patent vessel in 3 patients (14%) while 4 patients (19%) presented with a lesion in a conductance vessel with a minor interventional risk. Evaluation of the angiograms preceding the interventional attempt demonstrated that of the lesions eventually treated 14 were old lesions and 7 were new lesions, the latter regarded as a sign of progression of atherosclerosis. Duration of the intervention in this high-risk group was 66 ± 42 minutes (range 25–206 minutes) as compared to the average result of 34 ± 20 minutes in 2315 patients who underwent PCI in our catheterization laboratories in that year. The use of contrast media amounted to 247 ± 209 ml (range 50–750 ml) as compared to the average use of 182 ± 116 ml while radiation was used with an exposure of 14267 ± 9573 μgy/m^2 ^in comparison to 6987 ± 6481 μgy/m^2 ^in the general patient group. An average of 1.6 ± 0.9 guiding catheters (range 1–4 guides), 2.0 ± 1.4 PCI guiding wires (range 1–6 wires), 1.8 ± 1.0 balloons (range 1–4 balloons) and 1.6 ± 1.3 stents (range 0–5 stents) were used. Of 21 lesions treated 15 (71.4%) could be revascularized successfully.

#### Angulated lesions

**Figure 1:**
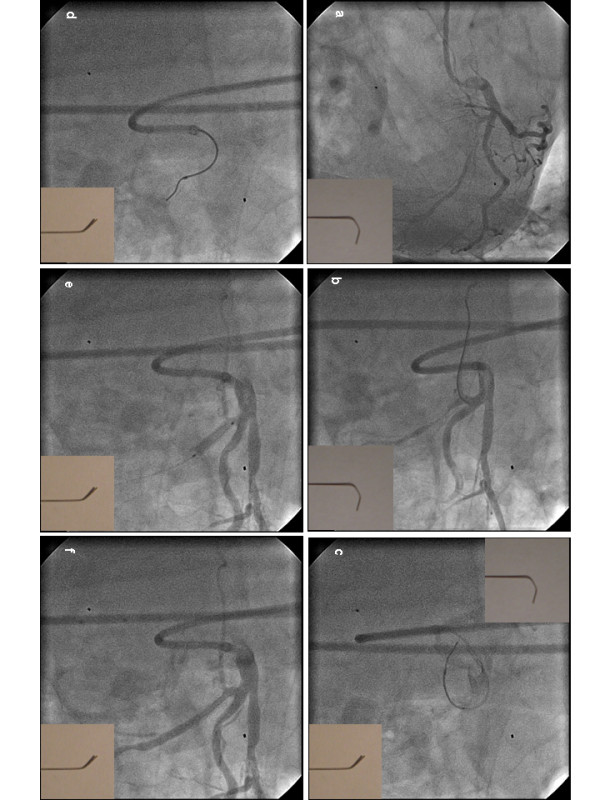
**"Park'n Ride"-Maneuver**. (a) Angulation of the circumflex artery <90°, early branching and tight stenosis in the proximal part of the circumflex artery. (b) Fish-hook shape of a Traverse™ PCI wire (bottom right) and passing of the wire into an atrial side branch of the circumflex artery with a Transit™ infusion catheter ("turn the corner"). (c) The infusion catheter rests in the atrial side branch ("park") while the flexible fish-hook shaped wire is exchanged against a more stable Balance Middle Weight™ wire with a flat and short-tipped shape ("steer"). (d) The wire is retracted into the infusion catheter so that only 2–3 mm of the wire are free in the vascular lumen. The infusion catheter is then pulled back with the wire ("back-up"). As soon as the wire snaps into the lumen of the main vessel it is steered forward through the tight stenosis ("ride"). (e) After balloon inflation a stent is placed. (d) complete opening of the circumflex artery, no dissection, atrial side-branch is preserved.

Skillful wiring was essential for success in extremely angulated stenoses. A soft wire with a core-to-tip design (Traverse™ Guidant, Santa Clara CA, USA) was often used for access to the coronary periphery to be later exchanged via an over-the-wire system (either balloon or infusion catheter) to a more stable wire (Balance Middle Weight™, Balance Heavy Weight™ or Extrs'port™ Guidant, Santa Clara CA, USA) for better stability. In extremely angulated lesions a soft infusion catheter (Transit™ 2.5 F, Cordis, Miami, FL, USA) was used for passing the wire through the vessel. Treating these patients we developed a maneuver we termed "park'n ride maneuver" (Figure 1). In patients with an angulation of the circumflex artery <90° it solves the problem of extreme bending plus a long tip of the PCI guide wire ("fishing hook") necessary for entering the circumflex artery and the soft bending of the wire of <45° plus a short tip needed to pass the wire through a tight proximal stenosis.

In one patient a high take-off right coronary bypass could be accessed with a 5F Multipurpose diagnostic catheter (Cordis, Miami, FL, USA) but the 7F Multipurpose interventional catheter did not fit due to a different angulation of the catheter. Therefore a "long'n soft glide change-over maneuver" was developed during the procedure. The diagnostic catheter was placed into the take-off of the right coronary bypass and a 3 meter PCI guide wire (Balance Middle Weight™, Guidant, Santa Clara CA, USA) was inserted via the diagnostic catheter into the periphery of the right coronary artery. Using an over-the-wire maneuver the diagnostic catheter was the exchanged against an interventional 7F multipurpose interventional catheter. Advancing the catheter over the thin wire particular care was taken to avoid loop formation of the wire in the ascending aorta. After insertion of the interventional catheter the PCI was performed in the usual fashion.

#### Chronic total occlusions

**Figure 2:**
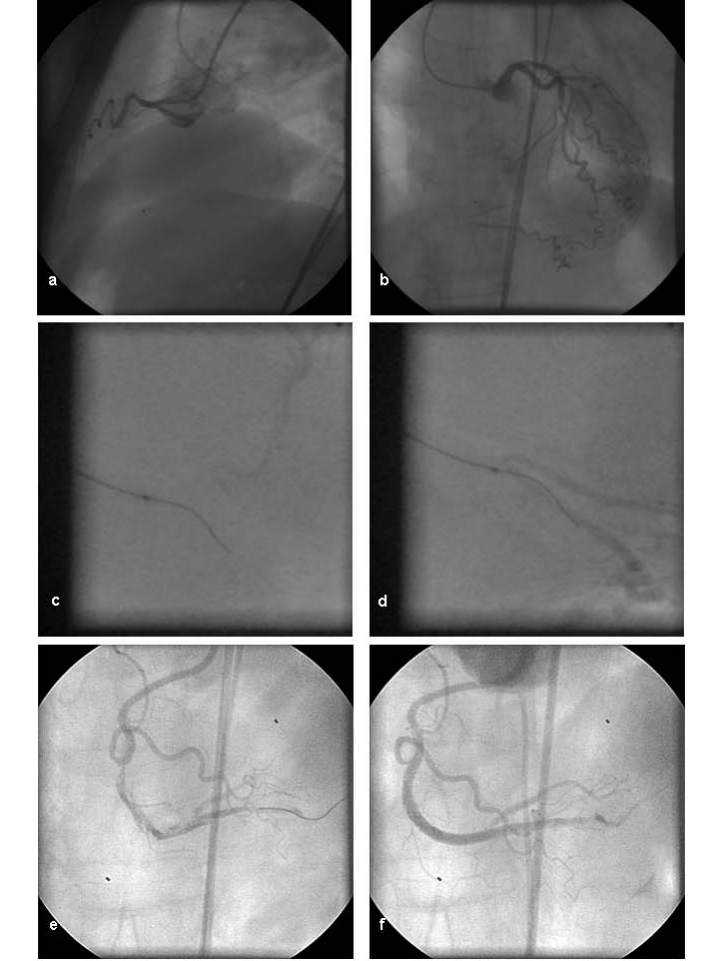
**Revascularization of the right coronary artery with double-injections**. a) occluded right coronary artery, b) retrograde filling of the right coronary artery via injection into the left coronary artery, c) passage of the PCI guiding wire via an over-the-wire system into the periphery of the right coronary artery, d) verification of intraluminal wire placement via injection into the left coronary artery and retrograde filling, e) opened right coronary artery with multiple dissections, f) opened right coronary artery after stent placement.

In chronic total occlusions stable back-up was of critical relevance for interventional success; Amplatz Left configurations were often employed for the right coronary artery. For access to the occlusion a low profile over-the-wire balloon (1.5 × 9 mm, OTW Maverick™, Boston Scientific, Maple Grove, MN, USA) was used with soft wire (Balance Middle Weight™). Passage through the chronic total occlusion was primarily tried with hard wires (XT 100™ – XT400™, Guidant, Santa Clara CA, USA) as tactile feel was regarded superior to other wires. In cases where this approach did not yield success in particular when catheter back-up was poor more hydrophilic wires (PT Graphix Intermediate™ Boston Scientific, Maple Grove, MN, USA or Shinobi™, Cordis, Miami, FL, USA) were tried. In some patients in whom the distal end of the occlusion was not visible double injections for proper targeting of the wire were performed (Figure 2).

#### Last patent vessel

In one patient with last patent vessel, large right coronary artery venous bypass, intraaortic ballon pumping was started prior to the procedure and was maintained for 8 hours after successful stenting of the proximal part of the bypass. Using a "hit-and-run approach" with primary stenting and an inflation time of 10 seconds drop of systolic arterial blood pressure from 100 mmHg to 65 mmHg could be kept to a time of 35 seconds without the use of catecholamines.

### Cardiac surgery

Complete hemodynamic work-up yielded indications for cardiac surgery apart from CABG. One patient who had been previously treated by us was than treated by his local cardiologists. He was then again referred to us for further treatment after three high-risk PCIs had failed in another center where he had been again classified to suffer form refractory angina pectoris. Complete work-up revealed a progression of valvular heart disease with severe low-gradient aortic stenosis (mean gradient 40 mmHg, orifice area 0.48 cm^2^, left ventricular ejection fraction 21%) who successfully underwent surgery. In another patient who presented with severe heart failure and a left ventricular ejection fraction of 34% it was decided to implant a resynchronization device.

### Outcome

**Figure 3:**
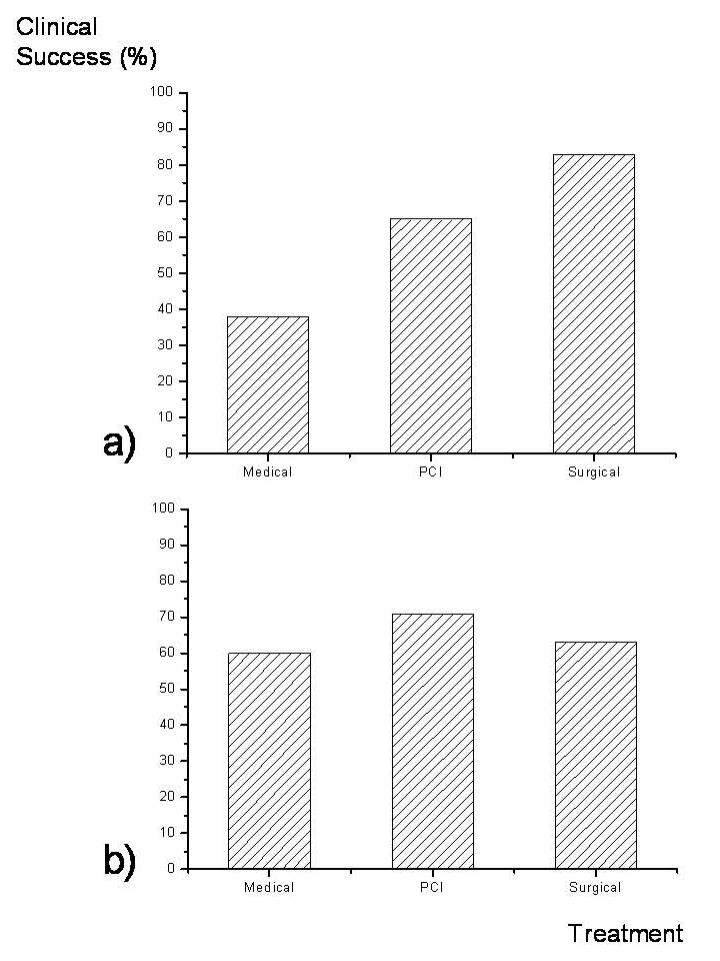
**Clinical success defined a reduction of clinical symptoms to Angina Class ≤II according to the criteria of the Canadian Cardiovascular Society for >30 days after begin of treatment based on a) primary-intention-to-treat in the medical (n = 8), PCI (n = 20) and surgical (n = 6) group and after b) cross-over with a medical (n = 5), PCI (n = 21) and surgical (n = 8) group (medical to PCI n = 2; medical to surgical n = 1; PCI to surgical n = 1)**.

Follow-up amounted to 9 ± 5 months (range 1–18 months). Based on an intention-to-treat clinical success as defined by a reduction to angina class II or lower for more 30 days following revascularization was achieved in 38% in the medical, in 63% in the PCI and in 83% in the surgical group. After cross-over 20 of 29 patients treated invasively (69%) improved clinically (Figure 3). Clinical success was achieved in 71.4% of the 21 patients treated with PCI and in 5 of 8 patients who underwent surgical treatment. 3 patients died from peri-interventional complications (2 after CABG, 1 after PCI). One patient died 2 months after the decision to treat medically.

**Figure 4:**
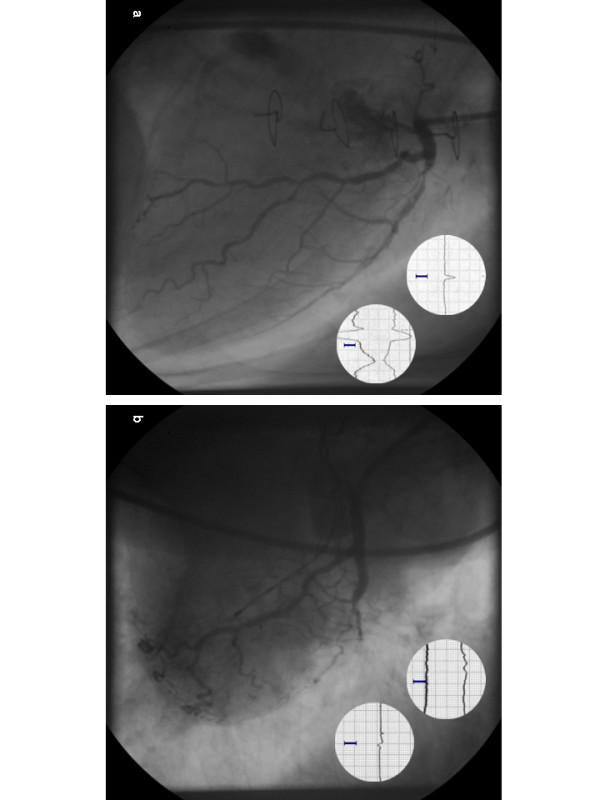
**(a) Chronic total occlusion of the right coronary artery with vital and ischemic myocardium of the posterior wall (scintigraphy, data not shown)**. b) Retrograde filling of the right coronary artery via injection into the left coronary artery; note simultaneous placement of the catheter in the right coronary and left coronary artery respectively in (a) and (b). (c) PCI wire in the distal right coronary artery. (d) Verification of intraluminal seating of the PCI wire in the right posterior descending artery with retrograde filling via the catheter in the left coronary artery. (e) Extensive dissection of the right coronary artery after balloon angioplasty. (f) Complete and stable revascularization after stent implantation.

One patient, a 79 year old lady in the group treated with PCI died as an immediate consequence of the procedure. She had a reversible scintigraphic perfusion defect of the anterolateral wall in a territory not supplied by a bypass with high grade stenosis of anterior descending artery deemed as the culprit vessel. She developed intermittent left bundle branch block already on injection of contrast media into the left coronary artery (Figure 4). During stent placement with an inflation time of 10 seconds she suddenly acquired complete atrioventricular block. Even though the angiographic result was good and a pacemaker lead was inserted fast via venous sheath placed at the beginning of the procedure she hemodynamically deteriorated rapidly and died within hours. We have since then changed our approach in patients with left bundle branch block during contrast media injection as we now place a pacemaker lead in the right ventricle before the intervention. One patient primarily treated medically was referred to coronary artery bypass operation for progressing unstable angina and died due to excessive hemorrhage presumably as a result of excessive adhesions secondary to previous CABG.

## Discussion

The study generates 3 major findings: (a) PCI can be performed effectively and safely in most patients with end-stage coronary artery disease previously diagnosed to suffer from refractory angina pectoris in end-stage coronary artery disease, (b) the interventional approach in these patients needs time, material and calls for interventional techniques which are not part of the standard repertoire and (c) new lesion formation may be responsible for renewed clinical symptoms.

The data presented show that primary interventional success in PCI can be achieved in almost two thirds of the patients with refractory angina pectoris and end-stage coronary artery disease. A major factor for interventional success in this series was surely the pooled experience of two senior cardiologists working on one case together. In particular during longer sessions or when progress slowed for example during recanalization of a CTO one investigator took over for some time or the rest of the procedure. The determination and perseverance of the whole team including assistance personnel can be derived from the average time of PCI which exceeds by far the average values for the general population treated in our catheterization laboratory. A major factor for a determined approach of the team was the fact that the long history of the patient's severe clinical symptoms was well know to the investigators and communicated to the rest of the team before the intervention. For example one patient primarily treated medically with an ejection fraction of 16%, angina pectoris grade III and a last patent vessel was seen 13 times at the outpatient clinic before it was decided together with the patient to try a high-risk approach.

Apart form the duration of intervention, the differentiated use of materials was surely another relevant factor for primary success. Differentiated use of PCI wires was essential for entering the coronary system in patients with extremely tortuous and angulated vessels. In particular the use of soft materials (infusion catheter + soft wire) was critical for access of the target vessel and passage through the stenosis while in the next step a more rigid and stabilizing wire was inserted in an over-the-wire maneuver for save deployment of treatment devices (balloons, stents). This approach drives up costs of the intervention in these patients. On the other hand that clinical success seen in these patients justifies the approach. Furthermore the learning curve included maneuvers newly developed during treatment of these high-risk patients. This led from the usual step-up approach using standard material first and then change to non-standard material to a primary top-step start where necessary which shortened duration and reduced use of material. Relevant was also the use of stents in particular in patients with CTOs as it not only stabilized the initial angiographic result but also raises the long-term patency rate [14-16]. As CTOs still have a relatively high rate of reocclusion we have made it a practice to follow-up these patients angiographically after 3–6 months irrespective of clinical symptoms. Even though the primary procedure was guided by clinical symptoms we feel that once a gate-way for coronary perfusion has been opened in these generally cirtically perfused hearts it should be kept open. The fact that the vast majority of patients treated with PCI could be also be improved symptomatically supports this approach irrespective of the occurrence of restenosis as it has been proven that the coronary lesion can be managed interventionally and should be further treated if necessary.

Interestingly one third of the patients presented with new lesions either in native coronary arteries or in bypass vessels. This has to be regarded as a sign of progression of atherosclerosis. These findings also signify the dynamics of myocardial perfusion and collateral formation as in the course of time another vessel may become causative for clinical symptoms in respect to the previous coronary status. At the initial time of diagnosis of refractory angina pectoris therapeutic measures may have led to a new balance of perfusion, for example as a result of symptomatic relieve allowing for more physical activity and collateral formation or metabolic adaptation resulting in long-term relieve after cessation of treatment as for example with long-term intermittent urokinase therapy. Natural progression of atherosclerosis may then disturb this balance leading to renewed symptoms. This is of particular clinical relevance because it means that the diagnosis of "refractory angina pectoris in end-stage coronary artery disease" is not a final one and angiographic reevaluation has always to be considered in the course of time or when symptoms progress.

The diagnosis of refractory angina pectoris in end-stage coronary artery disease may have two major consequences for the patient: a) the patient is labelled with a severe disease and b) he may be entered into experimental clinical studies which in turn may withhold established forms of therapy from the patient. Given the high rate of primary success PCI in patients with formerly refractory angina pectoris and end-stage coronary artery disease in this study the diagnosis of this distinct clinical symptom has to be seen critical. This was already pointed out in another, single- center study based on data from the early stent era where out of 117 patients referred to transmyocardial laser revascularization with presumably refractory angina pectoris 61% could be treated conventionally (45% medical, 16% invasive) leaving only 39% who underwent the non-established approach of transmyocardial laser revascularization [17]. Therefore careful work-up of these patients is mandated to rule out all conventional options for treatment of coronary artery disease before the diagnosis is established in the individual patient. This includes optimized medical treatment of coronary artery disease and heart failure [18] as well as consideration of interventional options like PCI and CABG by an experienced team.

A limitation of this study is that this is primarily an observational report and as such does not have the power of a randomized study. However, a well designed randomized study in these severely symptomatic patients might be impossible. The therapeutic decision will nearly always have to be done on an individual basis, and it will be difficult to control for probable placebo effects. We do not report long-term follow up data and can thus not judge about prognosis.

Also, the decision for a coronary intervention was made by one or two interventional cardiologists based on their extensive experience, and not by a multidisciplinary team. Not all patients underwent rigorous non-invasive testing for proving viable myocardium before the intervention. Although it should be essential to get as many information about myocardial viability as possible, this proofs to be difficult in this patient population. On the one hand these patients are highly symptomatic and any test involving exercise will show inconclusive results because of physical limitations. On the other hand these patients present with complex coronary artery disease with multiple stenosis and presumably a more generalized limitation of flow. This is frequently not visualized by SPECT. MRI might yield additional information but was not done in this study.

## Conclusion

Labelling a patient with a severe disease like refractory angina pectoris in end-stage coronary artery disease may have psychological consequences for the patient. Even though a cause-effect relationship between the cognitive conception of severe, potentially life-threatening coronary artery disease and the perception of intensity and frequency of angina pectoris in the patient is hard to establish, it seems likely. The reduction of clinical symptoms to grade II angina and lower in patients treated with PCI was considerable. From the interventional perspective it implies that a vessel critical for myocardial perfusion was targeted. This may be true but it has to be kept in mind though that an interventional procedure in patients with angina pectoris in itself can have a placebo effect of over 40% [19-21]. Subsequently clinical improvement may be a result of psychological factors involved at least in some of our patients. We are well aware that interventions performed in patients formerly classified to suffer from refractory angina pectoris and end-stage coronary artery disease may be more of symptomatic and psychological value rather than of prognostic relevance. Still we continue to pursue a primarily interventional strategy for treatment of patients with presumably refractory angina pectoris and end-stage coronary artery disease not only for the pathophysiological concept of myocardial ischemia but to assure the patient of a positive therapeutic perspective. After all, patients with presumably refractory angina pectoris and end-stage coronary artery disease suffer from a highly symptomatic and disabling disease and usually share with their treating physicians a long history of invasive treatment: they have a right to our continuous dedication living up to the best of our energies and skills.

## Competing interests

The authors declare that they have no competing interests.

## Authors' contributions

TJ coordinated the study, performed the statistical analysis and drafted the manuscript. AP coordinated the study, and drafted the manuscript. AAK coordinated the study and drafted the manuscript. MPH participated in the design and coordinated the study. FCS participated in the design and coordinated the study, and drafted the manuscript. All authors read and approved the final manuscript

## Pre-publication history

The pre-publication history for this paper can be accessed here:


